# Machine learning models for mortality prediction in critically ill patients with acute pancreatitis–associated acute kidney injury

**DOI:** 10.1093/ckj/sfae284

**Published:** 2024-09-11

**Authors:** Yamin Liu, Xu Zhu, Jing Xue, Rehanguli Maimaitituerxun, Wenhang Chen, Wenjie Dai

**Affiliations:** Department of Epidemiology and Health Statistics, Xiangya School of Public Health, Central South University, Changsha, Hunan, China; Department of Epidemiology and Health Statistics, College of Integrated Traditional Chinese and Western Medicine, Hunan University of Chinese Medicine, Changsha, Hunan, China; Department of Scientific Research, Xiangya Hospital, Central South University, Changsha, Hunan, China; Department of Epidemiology and Health Statistics, Xiangya School of Public Health, Central South University, Changsha, Hunan, China; Department of Nephrology, Xiangya Hospital, Central South University, Changsha, Hunan, China; Department of Epidemiology and Health Statistics, Xiangya School of Public Health, Central South University, Changsha, Hunan, China

**Keywords:** acute kidney injury, acute pancreatitis, eICU-CRD, in-hospital mortality, machine learning, MIMIC-IV

## Abstract

**Background:**

The occurrence of acute kidney injury (AKI) was associated with an increased mortality rate among acute pancreatitis (AP) patients, indicating the importance of accurately predicting the mortality rate of critically ill patients with acute pancreatitis–associated acute kidney injury (AP-AKI) at an early stage. This study aimed to develop and validate machine learning–based predictive models for in-hospital mortality rate in critically ill patients with AP-AKI by comparing their performance with the traditional logistic regression (LR) model.

**Methods:**

This study used data from three clinical databases. The predictors were identified by the Recursive Feature Elimination algorithm. The LR and two machine learning models—random forest (RF) and eXtreme Gradient Boosting (XGBoost)—were developed using 10-fold cross-validation to predict in-hospital mortality rate in AP-AKI patients.

**Results:**

A total of 1089 patients from the Medical Information Mart for Intensive Care-IV (MIMIC-IV) and eICU Collaborative Research Database (eICU-CRD) were included in the training set and 176 patients from Xiangya Hospital were included in the external validation set. The in-hospital mortality rates of the training and external validation sets were 13.77% and 54.55%, respectively. Compared with the area under the curve (AUC) values of the LR model and the RF model, the AUC value of the XGBoost model {0.941 [95% confidence interval (CI) 0.931–0.952]} was significantly higher (both *P* < .001) and the XGBoost model had the smallest Brier score of 0.039 in the training set. In the external validation set, the performance of the XGBoost model was acceptable, with an AUC value of 0.724 (95% CI 0.648–0.800). However, it did not differ significantly from the LR and RF models.

**Conclusions:**

The XGBoost model was superior to the LR and RF models in terms of both the discrimination and calibration in the training set. Whether the findings can be generalized needs to be further validated.

KEY LEARNING POINTS **What was known:**

- The occurrence of acute kidney injury (AKI) was associated with an increased mortality rate of acute pancreatitis (AP), indicating the importance of accurately predicting the mortality rate of critically ill patients with AP-AKI at an early stage.

**This study adds:**

- The eXtreme Gradient Boosting (XGBoost) model was superior to the logistic regression and random forest models in terms of both the discrimination and calibration in the training set with data from the Medical Information Mart for Intensive Care-IV and eICU Collaborative Research Database.

**Potential impact:**

- It is suggested to use the XGBoost model for better prediction of in-hospital mortality in critically ill patients with AP-AKI in clinical practice.

## INTRODUCTION

Acute pancreatitis (AP) is a leading cause of hospitalization for gastrointestinal diseases worldwide [1–3]. Numerous studies of different regions have reported a consistent increase in the incidence and hospitalization rates of AP [4–7]. Acute pancreatitis–associated acute kidney injury (AP-AKI) is one of the most common complications associated with AP [8–10]. The reported incidence of AP-AKI varies across studies, ranging from 7.9% to 69.3% [8, 11–13]. AKI is considered a primary cause of mortality in intensive care unit (ICU) patients with AP [12]. Previous studies have indicated that AKI could increase the mortality rate of AP patients by ≈5–6 times [4, 9] and its mortality rate was reported to be 80% in patients with severe AP [11, 12]. Therefore, accurately predicting the mortality rate of critically ill patients with AP-AKI at an early stage is of utmost importance.

Machine learning (ML) is a multidisciplinary field that combines mathematics with computer science. Its primary focus is on extracting knowledge from extensive datasets that encompass numerous variables and can handle intricate clinical information [14]. ML relies on mathematical algorithms to detect potential patterns within data, and its versatile algorithms, such as the commonly used algorithms eXtreme Gradient Boosting (XGBoost) and random forest (RF), become increasingly important in clinical data analysis. ML enables the development of robust risk models and enhances prediction capabilities, making it widely applicable in disease prediction, including the assessment of mortality rate in critically ill patients [14]. Several studies on ML models for predicting mortality rate or hospital prognosis in patients with AP or AKI have been reported [15–17]. However, currently there is a lack of research specifically focused on the development of an ML-based predictive model for in-hospital mortality rate in AP-AKI patients. Clinical severity scoring systems, such as the Sequential Organ Failure Assessment (SOFA) and Logistic Organ Dysfunction System (LODS), are commonly used to assess disease severity and mortality risk in critically ill patients [18–21]. Traditional logistic regression (LR) models are also frequently employed to predict mortality rate in critically ill patients. Nevertheless, methodological limitations in their clinical application have been indicated, including but not limited to low specificity and sensitivity, inadequate discriminative power and subpar prediction accuracy [20, 22, 23]. ML has shown potential advantages over traditional scoring systems and LR, exhibiting superior predictive performance [22, 24]. Hence it is necessary to develop an ML-based predictive model for assessing in-hospital mortality rate in critically ill AP-AKI patients, which can help accurately predict those with high risk of in-hospital mortality at an early stage in clinical practice.

Previous studies have identified some factors that may contribute to in-hospital mortality in AP-AKI patients [8, 25, 26]. Specifically, laboratory indicators included peak serum creatinine (SCr) >3 mg/dl and activated partial thromboplastin time (APTT) [25], comorbidities included circulatory failure and chronic diseases [4, 25, 26] and treatment requirements included the need for renal replacement therapy (RRT) and mechanical ventilation [4, 13, 25]. However, the sample size of these studies was limited, with a range from 44 to 287, and there is still a lack of comprehensive assessment of potential predictive factors [4, 8, 12, 13]. Therefore, this study aimed to develop and validate ML-based predictive models for in-hospital mortality rate in critically ill patients with AP-AKI by employing a large sample size and comprehensively assessing the role of demographic characteristics, laboratory indicators, vital signs, comorbidities and treatment requirements.

## MATERIALS AND METHODS

### Study design and population

This study was a retrospective cohort study that used data from three clinical databases: the Medical Information Mart for Intensive Care-IV (MIMIC-IV), the eICU Collaborative Research Database (eICU-CRD) and the clinical data repository from Xiangya Hospital of Central South University, Changsha, China. The MIMIC-IV (version 2.2) is a large, single-centre public database containing unidentified patient data from all ICU centres and emergency departments at Beth Israel Deaconess Medical Center (Boston, MA, USA) from 2008 to 2019 [27]. Similarly, the eICU-CRD (version 2.0) is a freely accessible multicentre critical care research database that encompasses data on >200 000 patients from 335 ICU centres across 208 hospitals in the USA between 2014 and 2015 [28]. The MIMIC-IV and eICU-CRD are both critical care databases, and the eICU-CRD was constructed following the MIMIC-IV and expanded the scope of research by including data from multiple healthcare institutions. Therefore, AP-AKI patients from these two databases might be homogeneous. To increase the statistical power for model development, AP-AKI patients from these two databases were combined to constitute the training set. AP-AKI patients from the ICU of Xiangya Hospital (January 2013–February 2023) were regarded as the external validation set. Xiangya Hospital is a nationally administered tertiary Grade A comprehensive hospital under the supervision of the National Health Commission of China.

To access the databases of MIMIC-IV and eICU-CRD, the Collaborative Institutional Training Initiative courses were completed (record ID: 58637411). The MIMIC-IV was approved by the Institutional Review Boards of Beth Israel Deaconess Medical Center and the Massachusetts Institute of Technology (Cambridge, MA, USA). Ethical approval for the eICU-CRD was not applicable because it was released under the Health Insurance Portability and Accountability Act (HIPAA) Safe Harbor provision. The external validation set was approved by the Ethics Committee of Xiangya Hospital (protocol 202403060). Due to the unidentified nature of the protected patient information in this study, written informed consent was not required. The study was reported in accordance with the Strengthening the Reporting of Observational Studies in Epidemiology statement [29].

Individuals who met the following criteria were included: a clinical diagnosis of AP and AKI and admission to the ICU. Individuals who met the following criteria were excluded: age <18 years, had an ICU stay of ≤24 hours and ≥25% missing data or outcome variable missing. For those with multiple admissions, only the first ICU admission was included in this study.

### Data collection

Data on demographic characteristics, laboratory indicators, vital signs, comorbidities and treatment requirements within the initial 24 hours following ICU admission were retrieved from three databases and were considered as potential predictors. Demographic characteristics included age, ethnicity and sex. Laboratory indicators included white blood cell (WBC) count, red blood cell (RBC) count, haemoglobin level, platelet count, haematocrit, neutrophil count, lymphocyte count, eosinophil count, basophil count, monocyte count, percentage of neutrophils (NEUT%), percentage of lymphocytes (LYMPH%), percentage of eosinophils (EO%), percentage of basophils (BAS%), percentage of monocytes (MONO%), mean corpuscular volume (MCV), mean corpuscular haemoglobin (MCH), mean corpuscular haemoglobin concentration (MCHC), red cell distribution width (RDW), blood urea nitrogen (BUN), SCr, albumin level, aspartate aminotransferase (AST) level, alanine transaminase (ALT) level, blood glucose (BG) level, potassium level, sodium level, chloride level, calcium level, phosphorus level, prothrombin time (PT), international normalized ratio (INR), and APTT. Vital signs included temperature, respiratory rate, heart rate, systolic blood pressure (SBP) and diastolic blood pressure (DBP). Comorbidities included coronary heart disease, diabetes mellitus, hypertension, hypertriglyceridaemia, malignancy, chronic pulmonary disease, chronic kidney disease (CKD) and sepsis. Treatment requirements included RRT, mechanical ventilation, glucocorticoid administration, diuretic administration and vasopressor administration.

The outcome variable of this study was in-hospital mortality, and relevant data were extracted from three databases. Due to the special social and cultural background in China, in-hospital mortality of the external validation set was defined as both mortality during the hospital and within 24 hours of discharge [30, 31].

### Statistical analysis

For normally distributed and homoscedastic continuous data, statistical descriptions were presented as the mean ± standard deviation (SD) and group differences were compared using the Student's *t*-test. Skewed data were described using median and interquartile range (IQR), with group differences assessed using the rank sum test. Categorical variables were presented as frequencies and percentages and group comparisons were conducted using the chi-squared test or Fisher's exact test. Missing values were imputed using the multiple imputation method by generating five datasets. Specifically, categorical missing values were combined using the mode of the generated data and continuous missing values were combined using the mean of the generated data as appropriate. *P*-values <.05 were considered statistically significant. All the statistical analyses were performed using R (version 4.3.1; R Foundation for Statistical Computing, Vienna, Austria).

### Model development

The training set included data from the MIMIC-IV and eICU-CRD. Multicollinearity of variables with statistical significance by between-group comparisons was assessed by the variance inflation factor (VIF), with a VIF value >5 considered to exhibit multicollinearity. The variable with the highest VIF was removed first, followed by recalculating the VIF for the remaining variables. This process was repeated until all variables had a VIF value <5. The Recursive Feature Elimination (RFE) algorithm was used for feature selection to identify the optimal subset. In each iteration, the least important feature was removed and the model was rebuilt. This process was repeated until the optimal feature subset was found. Finally, the optimal subset was selected as the predictors for the model.

For the XGBoost model, grid search was used and the model parameters were constantly tuned [32, 33], and for the RF model, the parameters were tuned one by one [34, 35]. To address the issue of class imbalance (death/survival), random oversampling was employed. Ten-fold cross-validation was used to develop the LR and ML models based on the selected predictors to predict in-hospital mortality rate among AP-AKI patients.

### Model evaluation and validation

The external validation set included data from the ICU of Xiangya Hospital. The discrimination of the predictive models in the training and external validation sets were assessed by the area under the curve (AUC), Youden index, accuracy (ACC), sensitivity, specificity, F1 score, positive predictive value (PPV) and negative predictive value (NPV). An AUC value of 0.7–0.8 and >0.8 is considered acceptable and satisfactory, respectively [36]. The Delong's tests were used to compare whether the differences in the AUC values across different models were statistically significant and Bonferroni's corrections were used for multiple comparisons. The models’ calibration was evaluated by the calibration curves and Brier scores. The Brier score ranges from 0.00 to 1.00, with closer to 0.00 representing better calibration.

For the predictive model with the highest performance in the training set, a variable importance ranking plot was generated to identify the most influential features. The Shapley Additive Explanations (SHAP) method, which is based on the Shapley value principle from game theory and analyses the contribution and contribution direction of each feature to the model's prediction, was employed to provide further explanation. The generalizability of the predictive model was evaluated based on the findings of the external validation set.

## RESULTS

### Characteristics of the study population

**Figure 1:**
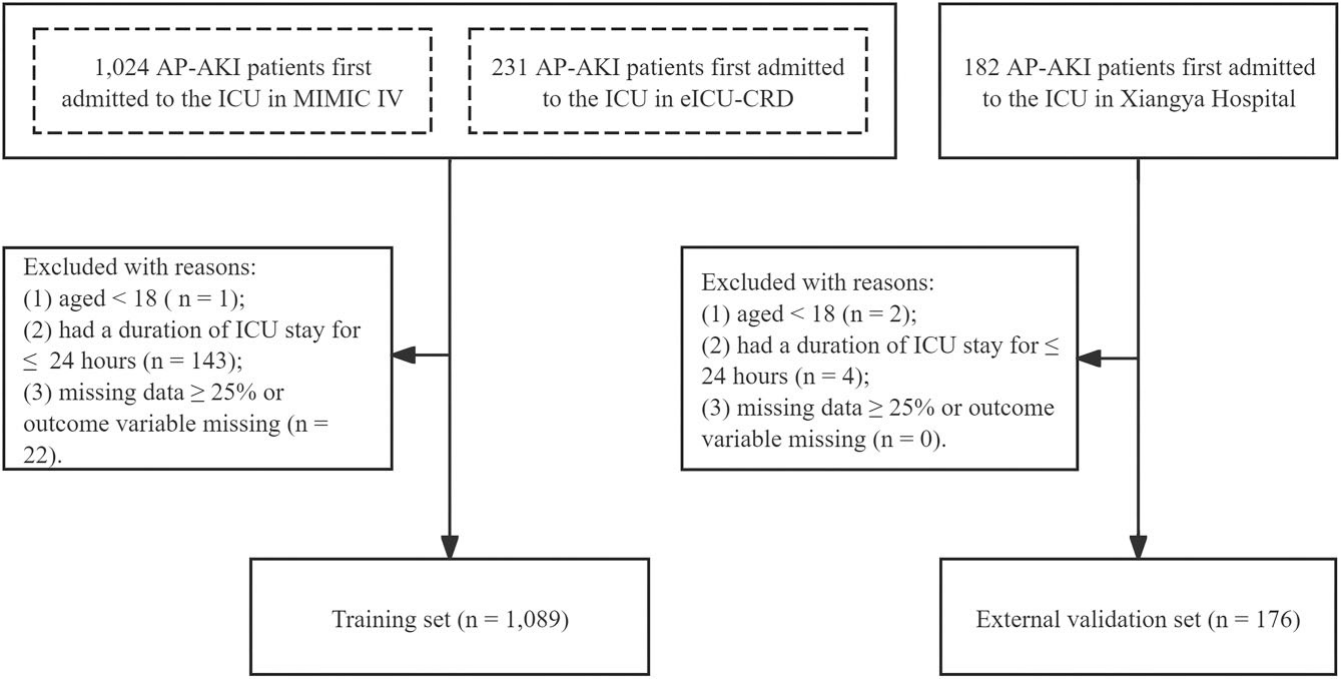
Flow chart of the study population.

A total of 1089 eligible records with AP-AKI were extracted from the MIMIC-IV and eICU-CRD and a total of 176 eligible AP-AKI patients from Xiangya Hospital were included in this study (Fig. 1). Thus the training and external validation sets consist of 1089 and 176 individuals, respectively. The in-hospital mortality rate of the training and external validation sets was 13.77% and 54.55%, respectively.

Table 1 shows the characteristics of the training and external validation sets. The patients in the training and external validation sets were ages 61.47 years (IQR 49.23–73.65) and 53.91 years (SD 1.12), respectively. Additionally, the proportion of females in the training and external validation sets was 45.27% and 24.43%, respectively. Additionally, the training and external validation sets differed significantly in laboratory indicators, including RBC, haemoglobin, platelet, haematocrit, lymphocytes, eosinophils, basophils, NEUT%, LYMPH%, EO%, RDW, BUN, SCr, AST, ALT, BG, sodium, calcium, INR and APTT; vital signs, including temperature, respiratory rate, heart rate and DBP; comorbidities, including coronary heart disease, hypertension, hypertriglyceridaemia, malignancy and chronic pulmonary disease; and treatment requirements, including RRT, mechanical ventilation and glucocorticoids (all *P* < .05).

**Table 1:** Characteristics of the training and external validation sets.

Characteristics	Training set (*n* = 1089)	External validation set (*n* = 176)	*P*-value
Demographics			
Age (years)	61.47 (49.23–73.65)	53.91 ± 1.12	<.001
Female, *n* (%)	493 (45.27)	43 (24.43)	<.001
Ethnicity, *n* (%)			<.001
White	745 (68.41)	0 (0.00)	
Black	130 (11.94)	0 (0.00)	
Asian	30 (2.75)	176 (100.00)	
Hispanic	42 (3.86)	0 (0.00)	
Other/unknown	142 (13.04)	0 (0.00)	
Laboratory indicators			
WBC (×10^9^/l)	12.30 (8.40–18.10)	13.15 (8.53–17.50)	.572
RBC (×10^12^/l)	3.74 (3.13–4.35)	3.00 (2.49–3.91)	<.001
Haemoglobin (g/dl)	11.20 (9.40–13.10)	9.25 (7.60–11.60)	<.001
Platelet (10^9^/L)	196.00 (133.00–279.00)	151.50 (105.75–213.00)	<.001
Haematocrit (%)	34.54 ± 0.24	27.85 (23.08–35.08)	<.001
Neutrophil (×10^9^/l)	10.04 (6.51–15.07)	11.20 (6.90–16.10)	.218
Lymphocyte (×10^9^/l)	0.99 (0.63–1.56)	0.80 (0.50–1.28)	<.001
Eosinophil (×10^9^/l)	0.06 (0.01–0.14)	0.00 (0.00–0.10)	<.001
Basophil (×10^9^/l)	0.02 (0.00–0.05)	0.00 (0.00–0.04)	<.001
Monocyte (×10^9^/l)	0.56 (0.34–0.94)	0.60 (0.30–1.08)	.186
NEUT%	81.44 (74.67–87.40)	86.15 (81.6–91.05)	<.001
LYMPH%	9.00 (5.00–14.00)	6.70 (3.93–10.80)	<.001
EO%	0.50 (0.10–1.10)	0.20 (0.10–0.80)	<.001
BAS%	0.20 (0.04–0.40)	0.20 (0.10–0.40)	.092
MONO%	5.00 (3.00–7.00)	5.10 (3.20–7.88)	.104
MCV (fl)	92.00 (87.65–98.00)	91.65 (89.60–95.78)	.999
MCH (PG)	30.50 (28.80–32.10)	30.50 (29.40–31.50)	.737
MCHC (g/dl)	33.00 (31.90–34.00)	33.10 (32.12–33.84)	.236
RDW (%)	14.90 (13.80–16.50)	14.60 (13.90–15.70)	.011
BUN (mg/dl)	23.00 (14.00–39.00)	43.54 (28.39–70.60)	<.001
SCr (µmol/l)	106.08 (70.72–193.15)	255.70 (141.68–414.10)	<.001
Albumin (g/dl)	3.00 (2.50–3.50)	2.92 ± 0.03	.072
AST (U/l)	67.00 (33.00–156.50)	55.40 (30.10–109.15)	.023
ALT (U/l)	46.00 (23.00–119.50)	26.35 (14.25–64.25)	<.001
BG (mmol/l)	7.50 (5.86–10.50)	10.20 (7.38–13.28)	<.001
Potassium (mmol/l)	4.10 (3.70–4.70)	4.05 (3.76–4.76)	.912
Sodium (mmol/l)	138.00 (134.00–141.00)	142.21 ± 0.53	<.001
Chlorine (mmol/l)	103.00 (99.00–108.00)	103.05 ± 0.56	.953
Calcium (mmol/l)	2.03 (1.85–2.18)	1.97 (1.76–2.10)	<.001
Phosphorus(mmol/l)	1.13 (0.87–1.45)	1.04 (0.66–1.52)	.072
PT (s)	14.70 (12.90–17.70)	15.30 (13.70–17.10)	.161
INR	1.30 (1.10–1.60)	1.26 (1.14–1.42)	.007
APTT (s)	31.00 (27.48–38.50)	38.25 (33.05–46.00)	<.001
Vital signs			
Temperature (°C)	36.78 (36.44–37.22)	37.00 (36.60–38.00)	<.001
Respiratory rate (breaths/min)	20 (17–25)	23 (20–30)	<.001
Heart rate (beats/min)	97 (83–114)	114.93 ± 1.60	<.001
SBP (mmHg)	123 (105–144)	126.16 ± 1.96	.599
DBP (mmHg)	68 (57–82)	74 (64–83)	.015
Comorbidities, *n* (%)			
Coronary heart disease	219 (20.11)	17 (9.66)	.001
Diabetes mellitus	341 (31.31)	48 (27.27)	.281
Hypertension	587 (53.90)	48 (27.27)	<.001
Hypertriglyceridaemia	249 (22.87)	85 (48.30)	<.001
Malignancy	101 (9.27)	4 (2.27)	.002
Chronic pulmonary disease	219 (20.11)	6 (3.41)	<.001
CKD	214 (19.65)	30 (17.05)	.416
Sepsis	558 (51.24)	93 (52.84)	.693
Treatment requirements, *n* (%)			
RRT	150 (13.77)	57 (32.39)	<.001
Mechanical ventilation	351 (32.23)	94 (53.41)	<.001
Glucocorticoid	191 (17.54)	15 (8.52)	.003
Diuretic	238 (21.85)	43 (24.43)	.445
Vasopressor	384 (35.26)	56 (31.82)	.373
Outcome variable, *n* (%)			
In-hospital mortality	150 (13.77)	96 (54.55)	<.001

Values are presented as median (IQR) or mean ± SD unless stated otherwise.

### Predictor selection

The final optimal subset of features identified eight variables: age, neutrophils, RDW, BUN, albumin, SBP, RRT and vasopressor. These eight variables were considered as the predictors for developing the in-hospital mortality predictive models.

### Model development

**Figure 2:**
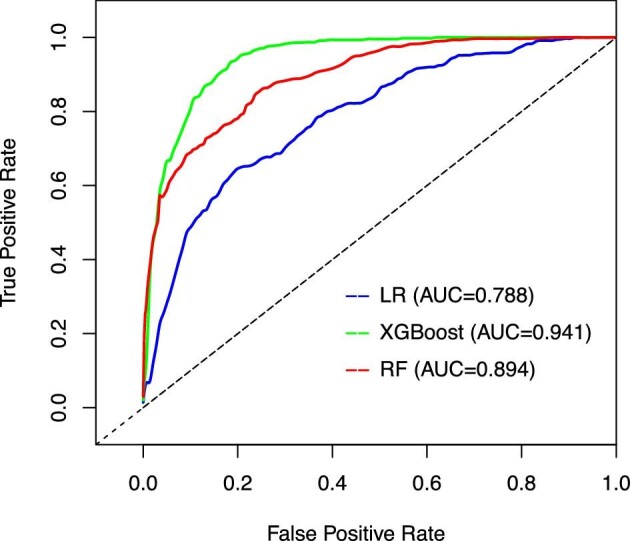
The receiver operating characteristics curves of the training set.

Table 2 shows the results for the discrimination of the LR, RF, and XGBoost models in the training set. Compared with the AUC values of the LR model [0.788 (95% CI 0.767–0.808)] and the RF model [0.894 (95% CI 0.880–0.908)], the AUC value of the XGBoost model [0.941 (95% CI 0.931–0.952)] was significantly higher (both *P* < .001) in the training set (Fig. 2).

**Table 2:** Discrimination indicators of the predictive models in the training and external validation sets.

Models	AUC (95% CI)	Youden index	ACC (%)	Sensitivity (%)	Specificity (%)	F1 score	PPV (%)	NPV (%)
Training set								
LR	0.788 (0.767–0.808)	0.45	72.42	64.64	80.19	0.70	76.54	69.40
XGBoost	0.941 (0.931–0.952)	0.75	87.49	93.50	81.47	0.88	83.46	92.62
RF	0.894 (0.880–0.908)	0.61	80.51	85.62	75.40	0.81	77.68	83.99
External validation set								
LR	0.691 (0.613–0.769)	0.23	59.66	40.62	82.50	0.52	73.58	53.66
XGBoost	0.724 (0.648–0.800)	0.19	56.82	32.29	86.25	0.45	73.81	51.49
RF	0.677 (0.599–0.756)	0.23	59.66	42.71	80.00	0.54	71.93	53.78

**Figure 3:**
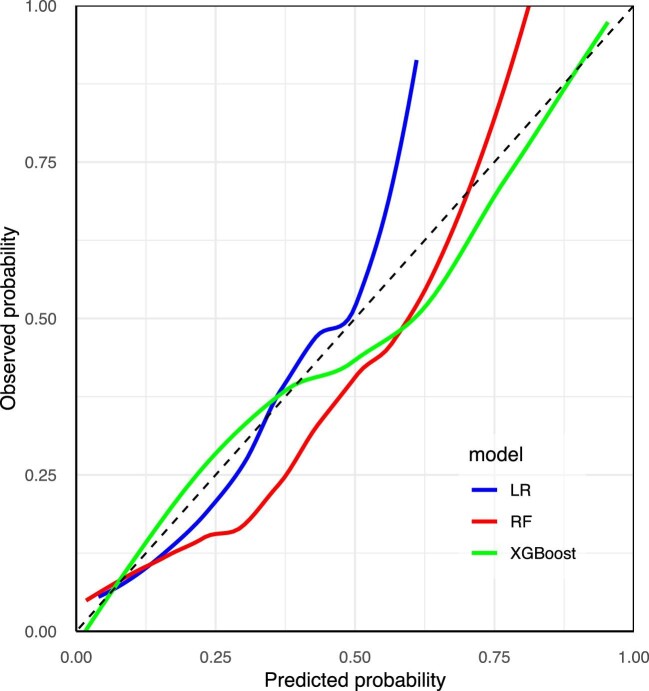
The calibration curves of the training set.

**Figure 4:**
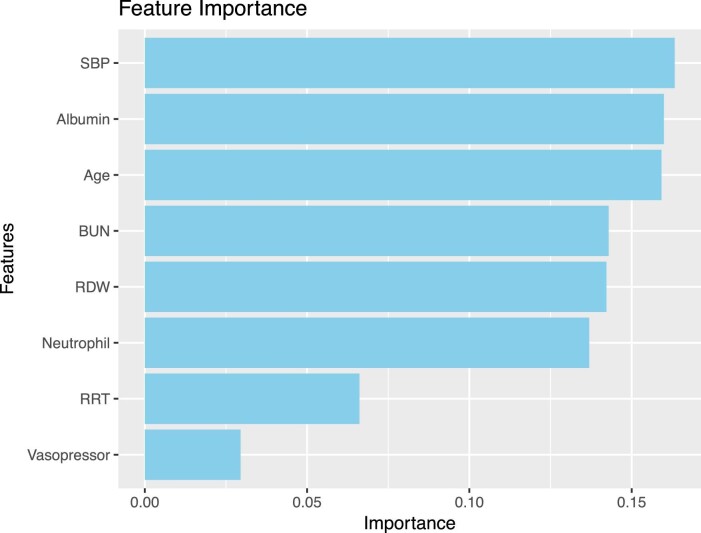
The feature importance rankings in the XGBoost model of the training set.

**Figure 5:**
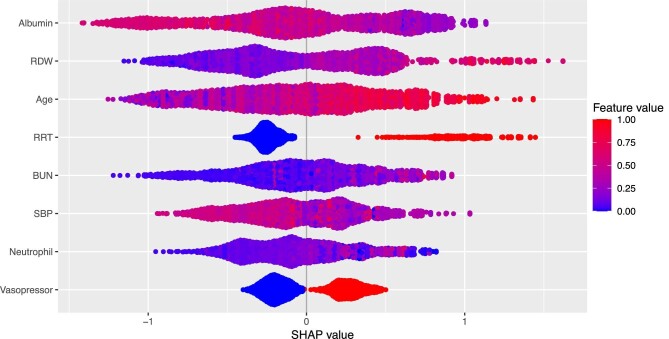
The importance of each feature based on the SHAP values of the training set. Each line in the *y*-axis represents a feature and the *x*-axis represents the SHAP value of the feature. Each dot represents a participant and the feature value is indicated by the colour, with red indicating a higher value and blue indicating a lower value. The positive associations between the feature and SHAP values suggest risk factors of in-hospital mortality rate, whereas the negative associations suggest protective factors of in-hospital mortality rate.

Figure 3 shows the calibration curves of the established models in the training set. The Brier scores of the LR, RF and XGBoost models were 0.099, 0.066 and 0.039, respectively. Figure 4 shows the feature importance rankings in the XGBoost model and Fig. 5 shows the importance of each feature based on its SHAP values.

### Model validation

**Figure 6:**
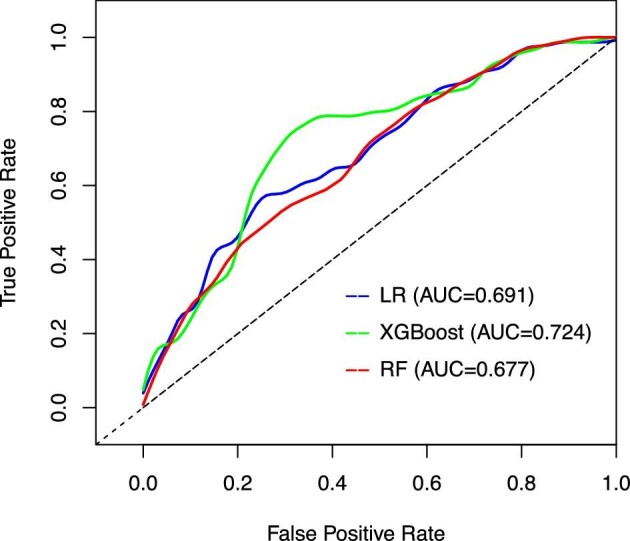
The receiver operating characteristics curves of the external validation set.

**Figure 7:**
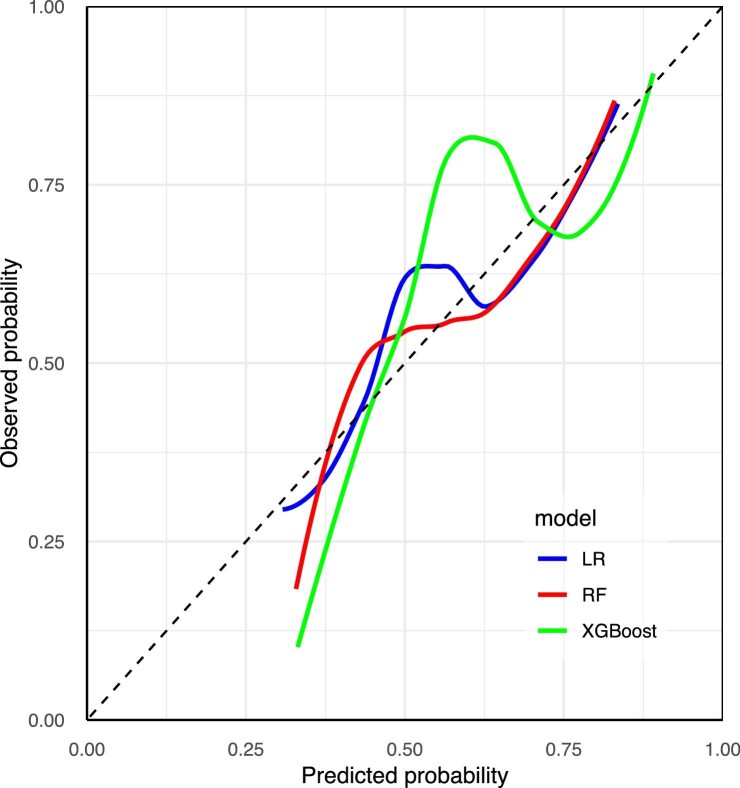
The calibration curves of the external validation set.

The results for the discrimination of the LR, RF and XGBoost models in the external validation set are also shown in Table 2. The performance of the XGBoost model was acceptable, with an AUC value of 0.724 (95% CI 0.648–0.800). However, it did not differ significantly from the LR (*P* = .898) and RF models (*P* = .206) in the external validation set (Fig. 6). Figure 7 shows the calibration curves of the established models in the external validation set. The Brier scores of the LR, RF and XGBoost models were 0.221, 0.224 and 0.217, respectively.

## DISCUSSION

This study developed two ML-based predictive models for in-hospital mortality rate in critically ill patients with AP-AKI using eight predictors by comparing their performance with the traditional LR model in a training set with a large sample size. This study found that the XGBoost model was superior to the other two models for both discrimination and calibration. The finding was further validated using an external cohort with a limited sample size, and a similar tendency was observed in the external validation set. To the best of our knowledge, this is the first study to develop ML-based models to predict in-hospital mortality rate of critically ill patients with AP-AKI.

XGBoost is a gradient boosting tree algorithm that leverages efficient algorithms and optimization techniques to construct powerful and efficient ML models. Consequently, the XGBoost model offers several advantages, including high accuracy and scalability, making it suitable for a wide range of regression and classification problems. The majority of prior work on comparing the performance between the XGBoost and traditional LR models showed the superiority of the XGBoost model, especially in clinical prediction [37, 38]. For instance, in a study on ML for predicting AKI in sepsis patients, the XGBoost model exhibited the best predictive performance among nine models in terms of discrimination, calibration and clinical applicability [38]. Similarly, in another study predicting 28-day mortality rate in sepsis-associated AKI patients, the XGBoost model outperformed traditional scoring systems and other ML models, demonstrating superior predictive performance [16]. Consistently, this study found that the XGBoost model was superior to the LR and RF models for both discrimination and calibration in the training set. Therefore, it is suggested to use the XGBoost model for better prediction of in-hospital mortality in critically ill patients with AP-AKI in clinical practice.

This study identified eight predictors—age, neutrophils, RDW, BUN, albumin, SBP, RRT and vasopressor—to develop the in-hospital mortality predictive models. Previous studies have indicated the role of age in predicting mortality among patients with severe illnesses [18, 25, 39, 40]. The association of advanced age with increased mortality rate can be attributed to age-related physiological decline, such as decreased immune function and organ dysfunction [41–43]. Therefore, AP-AKI patients with advanced age should be given special attention to reduce the mortality rate.

The predictive value of RDW and serum albumin concentration in predicting in-hospital mortality has been indicated in previous studies of critically ill patients [44–52]. Its underlying mechanisms have not been fully elucidated and it has become evident that RDW may be a non-specific parameter to provide effective risk stratification for patients with severe illnesses [44]. The neutrophil:lymphocyte ratio (NLR) is a marker of inflammation and physiological stress. The NLR is strongly correlated with early onset, progression and recovery in AKI, as well as the in-hospital and post-discharge mortality rates of these patients [53]. For example, a study found that high NLRs were associated with an increased risk of 30-day and 90-day mortality in AKI patients [54]. Additionally, BUN is an important blood test indicator of kidney function. A previous study found that the BUN:creatinine ratio was useful for risk stratification of AKI [55]. Another study indicated that the BUN:serum albumin ratio was a promising and easily obtainable biomarker that can serve as a prognostic predictor for AKI and in-hospital mortality in ICU patients with intracerebral haemorrhage [56]. Consistently, this study found similar results, indicating the possible value of neutrophils, RDW, BUN and albumin as indicators of in-hospital mortality rate in critically ill patients with AP-AKI.

In terms of vital sign measurements, a previous study found that patients with an SBP of 180–199 mmHg have a decreased risk of developing AKI compared with those with an SBP <180 mmHg [57]. Similarly, this study found protective effects of high SBP against in-hospital mortality in critically ill patients with AP-AKI. Additionally, this study found that treatment requirements of RRT and vasopressor were predictors for in-hospital mortality in critically ill patients with AP-AKI, which was consistent with previous studies in numerous populations and could be explained by the fact that those treated with RRT or vasopressor were more critical compared with their counterparts [13, 25, 31, 58]. Therefore, more medical resources should be allocated to those treated with RRT or vasopressor in the context of limited medical resources for critically ill patients with AP-AKI patients.

The strengths of this study included the retrospective cohort design with multiple ICU centres, the large sample size of the training set, the comprehensive assessment of possible predictors and the external validation of the established models, which significantly added credibility when interpreting relevant findings. However, some limitations should be acknowledged. First, this study used data from established databases. Therefore, the predictive value of factors that were not recorded was unknown. For example, the aetiology of pancreatitis, which was not considered in this study, may be associated with the in-hospital mortality of critically ill patients with AP-AKI. Similarly, this study considered factors collected within the initial 24 hours following ICU admission exclusively. Therefore, the predictive value of factors collected out of this time frame need to be further explored. Furthermore, the characteristics of the study population, the in-hospital mortality rate and the sample size of the external validation set varied greatly from the training set, which may affect the performance of the predictive models in the external validation set, although a similar tendency was observed. Therefore, the generalizability of the findings of the training set remains an issue and future validation is still needed.

## CONCLUSIONS

The XGBoost model was superior to the LR and RF models in terms of both the discrimination and calibration in the training set with data from the MIMIC-IV and eICU-CRD, and eight predictors (age, neutrophil, RDW, BUN, albumin, SBP, RRT and vasopressor) were identified. However, due to the differences in the characteristics of the study population, the in-hospital mortality rate and the sample size between the training and external validation sets, the generalizability of the findings of the training set remains an issue and future validation is still needed.

## Data Availability

The data that support the findings of this study are available from the corresponding authors upon reasonable request.
